# Metabolic Pathways, Enzymes, and Metabolites: Opportunities in Cancer Therapy

**DOI:** 10.3390/cancers14215268

**Published:** 2022-10-27

**Authors:** Rishabh Kumar, Anurag Mishra, Priyanka Gautam, Zainab Feroz, Sivakumar Vijayaraghavalu, Eviania M. Likos, Girish C. Shukla, Munish Kumar

**Affiliations:** 1 Department of Biochemistry, Faculty of Science, University of Allahabad, Prayagraj 211002, UP, India; 2 Department of Life Sciences (Zoology), Manipur University, Imphal 795003, MN, India; 3 Center for Gene Regulation in Health and Disease, Department of Biological, Geological, and Environmental Sciences, Cleveland State University, 2121 Euclid Avenue, Cleveland, OH 44115, USA

**Keywords:** cancer metabolism, glycolysis, fatty acid metabolism, amino acid metabolism, Warburg effect, microRNA, oncogenes, tumor suppressor genes, cancer therapeutics

## Abstract

**Simple Summary:**

Deregulated cellular metabolism is one of the major hallmarks of cancer. Cancer cells orchestrate abnormal metabolic reprogramming to satisfy high energy demands. The review focuses on the mechanics of the major metabolic pathways, significant intermediates, and associated enzymes that are altered by the oncogenic progression. The emphasis is laid on therapeutically targeting clinically relevant metabolic intermediates which are crucial to cancer cell survival, and proliferation. The clinical intervention of metabolic pathways, critical enzymes, and the intermediate, thus offers a distinct niche in cancer therapies.

**Abstract:**

Metabolic reprogramming enables cancer cells to proliferate and produce tumor biomass under a nutrient-deficient microenvironment and the stress of metabolic waste. A cancer cell adeptly undergoes a variety of adaptations in metabolic pathways and differential expression of metabolic enzyme genes. Metabolic adaptation is mainly determined by the physiological demands of the cancer cell of origin and the host tissue. Numerous metabolic regulators that assist cancer cell proliferation include uncontrolled anabolism/catabolism of glucose metabolism, fatty acids, amino acids metabolism, nucleotide metabolism, tumor suppressor genes, microRNAs, and many regulatory enzymes and genes. Using this paradigm, we review the current understanding of metabolic reprogramming in tumors and discuss the new strategies of cancer metabolomics that can be tapped into for cancer therapeutics.

## 1. Introduction

Due to the rising prevalence of cancer throughout the globe, it is of utmost importance to investigate the underlying processes that contribute to cancer development to combat this looming issue [1]. While a wide range of genetic, molecular, and cancer metabolism research has led to great advancements and landmark discoveries in this field, much remains to be learned about cancer metabolism. Otto Warburg established in the 1920s that cultured tumor tissues absorb glucose and generate lactate at high rates even when oxygen is present (aerobic glycolysis) [2,3]. The three metabolic features that define the Warburg effect are glucose intake, lactate secretion, and oxygen availability. Long before Warburg, Pasteur demonstrated that oxygen inhibits sugar fermentation, recognizing glucose-to-lactate conversion as an expected reaction to hypoxia. If this notion is applied to cancer, tumors may be hypoxic, and hypoxia may boost lactate generation in tumors, as it does everywhere. That, however, is not the Warburg effect. Even when there was adequate oxygen to convert glucose to CO_2_, which other tissues prefer and which we now know is far more productive in terms of ATP synthesis, the tumor samples took up glucose and converted it to lactate [4,5]. Because tumor cells demand a limitless source of energy, the ATP synthesis rate of glycolysis is significantly higher than that of oxidative phosphorylation, even though the efficiency of ATP production per molecule of glucose is substantially lower via glycolysis [6,7]. In reality, cancer cells can have dysregulated central metabolic pathways [8]. More crucially, new research suggests that cancer cells can inhibit anti-tumor immune responses by competing for and depleting vital resources, or by otherwise lowering the metabolic fitness of tumor-infiltrating immune cells [9,10]. Thus, this cancer hallmark provides a means for malignant cells to evade the body’s immune system, allowing cancerous cells to proliferate and cancer to progress to later stages [11,12].

The recent use of sophisticated technologies such as metabolomics, metabolic flux studies, and functional genomics has revealed that cancer cells and tumors exhibit diverse metabolic tendencies and dependencies. Metabolic phenotypes in tumors are both varied and flexible, resulting from the interplay of many distinct elements. These elements are often inherent to the cancer cell (e.g., cell lineage; differentiation state; somatically acquired mutations) while others are imposed by the microenvironment (e.g., nutrient milieu; interactions with extracellular matrix and stromal cells).

Furthermore, aberrant lipid metabolism, amino acid metabolism, mitochondrial biogenesis, and other cellular metabolism pathways have been associated with metabolic remodeling in cancer cells. Therefore, understanding how the metabolic phenotypes originate and evolve will necessitate an understanding of the effect of these components, as well as their proportional impact on tumor progression [13].

Physiological stress, such as the shortage of O_2_, is thought to be one of the key causes of the metabolic transition in tumor cells. Early tumor cells become hypoxic as they proliferate, but still, require blood and nutrients for continuous growth. Because of the decreased reliance on aerobic respiration, tumor metabolism is oriented toward glycolysis, balancing O_2_ demand with O_2_ supply. One key player during this adaptive response to low oxygen is hypoxia-inducible factor-1 (HIF-1), a transcription factor that accumulates and delivers O_2_ to tumor cells during hypoxia via enhancing angiogenesis, erythropoiesis, and rapid glycolysis, allowing cancer cells to thrive even under physiological stress [14,15,16].

Most tumor cells also exhibit significantly enhanced anabolism pathways, including aerobic glycolysis, glutaminolysis, fatty acid synthesis (FAS), and pentose phosphate pathway (PPP), compared to normal cells, which depend exclusively on oxidative phosphorylation (OXPHOS) to produce the energy needed for cellular homeostasis.

Additionally, glutamine is converted to glutamate by glutaminase and subsequently converted into alpha-ketoglutarate (α-KG) which replenishes and maintains the tricarboxylic acid (TCA) cyclic pathway [17].

Furthermore, in addition to producing phosphopeptides and ribonucleotides, the PPP is a key source of NADPH, a critical antioxidant for cellular redox adaptation [18,19,20].

**Figure 1:**
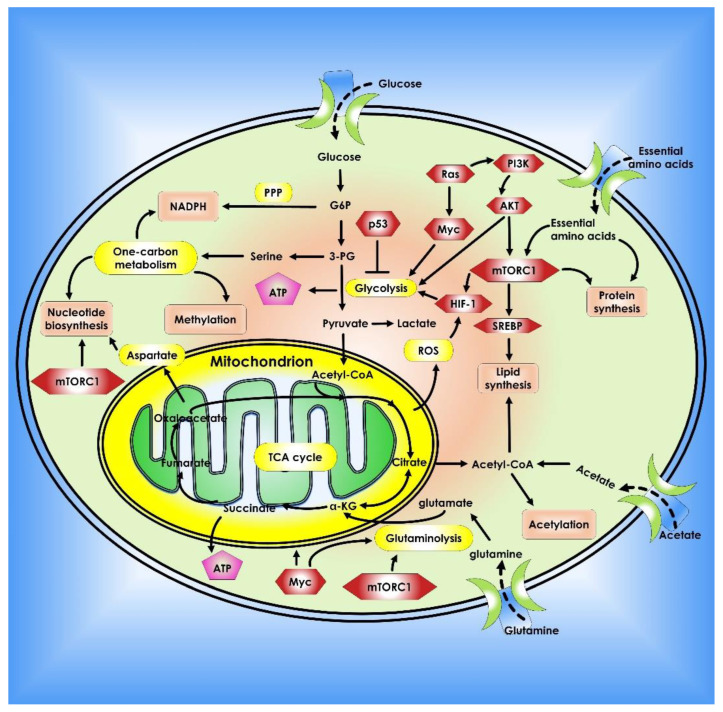
Metabolic pathways that control cancer’s ability to grow and spread. Nucleotide, protein, and lipid synthesis are induced by abnormal activation of mTORC1 in tumor cells. Further anabolism is promoted when tumor suppressors like *p53* are lost, or oncogenes are activated. Regulation of ROS, acetylation, and methylation by metabolism is one way that metabolic signaling is kept under control.

The reasons for cancer cells’ metabolic alterations are many and can be linked to both internal and external sources. Oncogenic and tumor suppressor signaling pathways, such as *HIF-1*, *p53*, and *Myc* genes, all contribute to the regulation of cancer cell metabolism [21]. Changes in oncogenes such as *Myc*, *Ras*, and *BRAF* have been linked to metabolic reprogramming [22,23,24]. *Myc* transcriptionally controls several metabolic enzymes involved in DNA synthesis and glycolysis, such as thymidylate kinase and lactate dehydrogenase A [25,26]. *Myc* is also implicated in the metabolic reprogramming of fatty acids, glutamine, proline, and nucleic acids, either directly or indirectly via various microRNAs (miRNAs) [26,27,28,29]. Overall, altered or dysregulated cellular metabolism is an extensive process that requires the use of several different pathways and factors that contribute to cancer development. Thus, investigating the reprogramming of energy metabolism might reveal underlying molecular processes in cancer and aid in the development of new methods for early detection and treatment (Figure 1).

## 2. Glucose Metabolism in Cancer Cells

Major metabolic pathways, including the glycolysis pathway, the PPP, and the serine synthesis pipeline (SSP) in the cytoplasmic compartment, as well as the TCA cycle in the mitochondria, are all fundamental to glucose metabolism. Glycolysis is an important pathway for glucose metabolism that results in the synthesis of pyruvate, which is converted into lactate for extracellular secretion or enters the TCA cycle in the mitochondria. The glycolysis is also linked to the PPP and the serine production process via its metabolic intermediate. Furthermore, the hexosamine biosynthetic pathway is branched from the glucose metabolism pathway via fructose-6-phosphate and contributes to protein modification by producing Uridine diphosphate N-acetylglucosamine (UDP-GlcNAc) as a byproduct. In addition, it also regulates growth factor-induced glucose and glutamine metabolism. Recent findings indicate that these pathways have been transformed or reconfigured in cancer cells [30].

Cancer cells favor aerobic glycolysis for ATP synthesis while keeping OXPHOS activity for the following reasons:

(i) Glycolysis is more conducive to cancer development. Because cancer cells proliferate faster than normal tissues, they require not just energy but also metabolic intermediates for macromolecule production. Many intermediates from glycolysis and the shortened TCA cycle can be utilized to synthesize macromolecules necessary for cancer development and proliferation, including nucleic acids, lipids, and proteins [31,32].

(ii) Overly efficient ATP products may be harmful to cancer cells. ADP is turned into ATP when cancer cells consume high-efficiency glucose. The high quantity of ATP inhibits phosphofructokinase 1 (PFK1), the rate-limiting p53 mutations enzyme in glycolysis, as well as pyruvate kinase 1 (PK1), resulting in glycolysis inhibition [33].

Although glycolysis produces less ATP than OXPHOS, the rate of ATP synthesis in the former is faster, making it suitable for the energy demands of rapidly proliferating cells such as cancer and embryonic tissues [32]. In general, fast proliferation tissues rely heavily on glycolysis for ATP synthesis, whereas differentiated tissues rely more on OXPHOS [13,34].

(iii) Hypoxia is commonly seen in cancer tissues, and glycolysis gives malignancies an edge in this hypoxic environment [35,36]. Lactate is produced by glycolysis and is released into the extracellular space. An acidic microenvironment gives cancer tissues a growth advantage over normal tissues and promotes cancer cell invasion and metastasis [37,38]. Furthermore, lactic acidosis limits glycolysis and promotes aerobic respiration as a source of energy [38,39,40].

(iv) As mitochondrial OXPHOS decreases, there is less production of reactive oxygen species (ROS), which are deadly to cancer cells [39,41].

Although cancer cells may preserve OXPHOS activity, this does not imply that they do not have mitochondrial respiration abnormalities. Enhanced glycolysis in certain cancers is caused by mitochondrial dysfunction [42,43], including decreased expression of mitochondrial oxidative enzymes and transporters. A truncated TCA cycle, a decrease in the number of mitochondria per cell and a defective respiratory chain, an increase in natural inhibitors of mitochondrial ATP synthase, and a higher sensitivity of mtDNA to oxidative stress [44,45].

Furthermore, glycolysis increase is not just for ATP production but also biomass synthesis, including ribonucleotides [35] and amino acids, for proliferation and growth in a constantly shifting microenvironment with many material constraints, such as oxygen and nutrient deficiencies. Enhanced glycolysis might potentially be explored as a technique to lower ROS and hence oxidative stress in cancer cells [46,47].

Cancer cells promote the primary step in glucose metabolism by boosting glucose absorption and generating high-level expression of hexokinase 2 (HK2), which is already expressed in normal cells [35,48]. HK1 is the most common isoform found across most adult tissues, whereas HK2 is exclusively present in skeletal muscle, heart, and adipose tissues, however, it is also detected in embryonic tissues. HK2 expression, on the other hand, is elevated in cancer cells to boost glucose flow into multiple metabolic pathways [49,50]. PFK1 catalyzes the second committed phase of glycolysis, whereas pyruvate kinases catalyze the third committed step. F6P is converted into fructose-1,6-bisphosphate (F1,6BP) by PFK1, while phosphoenolpyruvate (PEP) is converted into pyruvate by pyruvate kinases. The first two committed actions use ATP, while the third committed step creates ATP. Except for the third and final committed phase (which creates ATP and pyruvate), cancer cells employ multiple strategies to enhance the flow of glucose in glycolysis. The last committed step is mitigated in part by employing the low-affinity pyruvate kinase M2 (PKM2) isoform to catalyze this reaction [51].

Modulation of the final dedicated step of glycolysis disperses metabolites into bifurcation routes such as the PPP and the SSP. It generates enough metabolic intermediates and augments the anabolic processes necessary for cell growth and proliferation. Although the final dedicated step (the conversion of PEP to pyruvate) is reduced, the following conversion of pyruvate to lactate is significantly enhanced, with the majority of the lactate being released. The two most abundant isozymes, lactate dehydrogenase A (LDH-A) and lactate dehydrogenase B (LDH-B) can form homotetramers or heterotetramers. Because LDH-A has a larger affinity for pyruvate and LDH-B has a higher affinity for lactate, LDH-A prefers the forward reaction while LDH-B prefers the reverse reaction. In cancer cells, LDH-A is the most abundant LDH [52,53]. Lactate can be taken up by neighboring stromal cells and utilized as an energy source to maintain development or to produce pyruvate, which is subsequently released by the stroma and taken up by cancer cells [54,55]. Cancer cells can employ extracellular lactate or pyruvate to maintain the TCA cycle and supply citrate and acetyl-CoA for fatty acid synthesis when glucose is scarce.

LDH-A catalyzes the enhanced pyruvate to lactate flow in cancer cells, generating NAD+ from NADH. In glycolysis, NAD+ is transformed into NADH, which is necessary to sustain a high flux of glucose metabolism.

**Figure 2:**
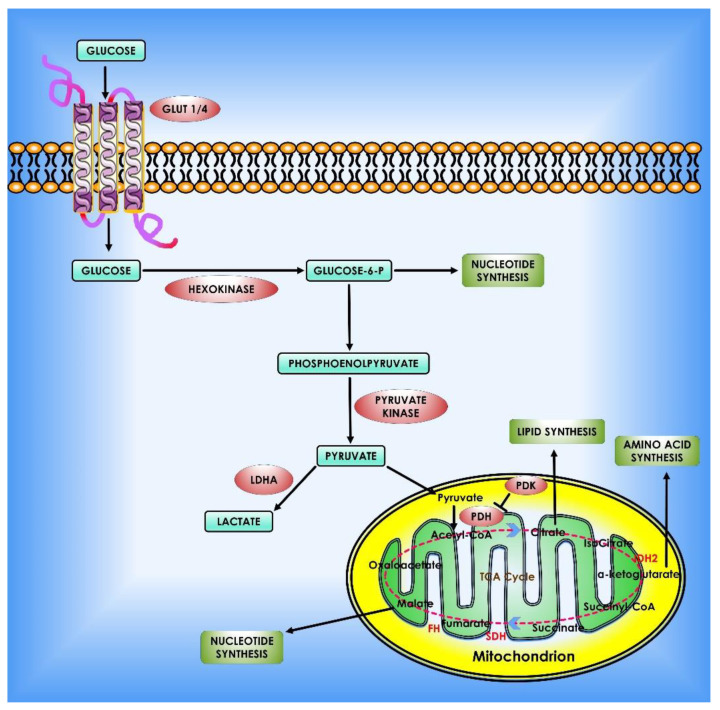
Glucose metabolism in cancer cells. When glucose enters the cell via glycolysis, it is phosphorylated by HK to glucose-6 phosphate, which is then converted by glycolysis to pyruvate in the cytosol. Under an aerobic environment, normal cells employ pyruvate dehydrogenase (PDH) to convert the majority of pyruvate to acetyl-CoA. The acetyl-CoA is subsequently oxidized via the TCA cycle, providing sources of ATP production. On the contrary, the molecular mechanisms of glucose consumption in cancer are switched from oxidative phosphorylation to glycolysis. Furthermore, cancer cells require the creation of new macromolecules to proliferate (for example, nucleic acids, lipids, and proteins). Critical enzymes which might be viable targets for cancer treatment are indicated in orange. TCA enzymes iso-citrate dehydrogenase 2 (IDH2), succinate dehydrogenase (SDH), and fumarate hydratase (FH) which are known to be mutated in cancer are depicted in red within the mitochondrion.

To reiterate, glycolysis is increased in cancer cells to nourish the branching pathways that produce nucleotides, amino acids, and fatty acids for anabolic processes [56]. Notably, numerous glycolytic enzymes’ activities are favorably and negatively controlled to maintain homeostasis. Cancer cells frequently use these regulatory mechanisms to meet their anabolic demands and adapt to different microenvironments (Figure 2).

## 3. Role of Oncogenes in Cancer Metabolism

**Figure 3:**
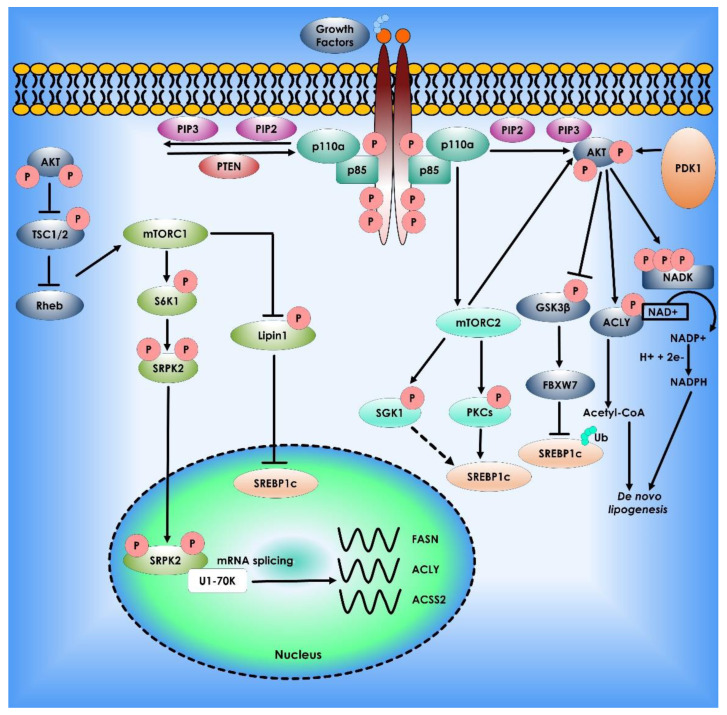
Akt mTOR signaling Pathway in Lipid Synthesis. The phosphatidylinositol-3-kinase (PI3K)/Akt and the mammalian target of rapamycin (mTOR) signaling regulate lipid metabolism. The most often dysregulated mechanism in cancer is PI3K signaling, which promotes growth, proliferation, and survival. When receptor tyrosine kinases are activated, PI3K is recruited to the plasma membrane and phosphorylates Phosphatidylinositol 4,5-bisphosphate (PIP2) to Phosphatidylinositol (3,4,5)-trisphosphate (PIP3). AKT binds to PIP3, allowing PDK1 and mTORC2 to activate. AKT stimulates lipogenesis directly by inhibiting Glycogen synthase kinase 3, activating ATP citrate lyase to make acetyl-CoA, and phosphorylating NADK to produce NADP+ for NADPH production. PI3K signaling is also intertwined with mTORC1 and mTORC2. mTORC1 controls lipogenesis by inhibiting lipin-1, a negative regulator of nuclear Sterol regulatory element-binding protein-1c, and activating the splicing factor Serine/threonine-protein kinase (SRPK2), boosting the production of lipogenic enzymes such as ATP citrate lyase (ACLY), Fatty acid synthase (FASN), and acetyl-CoA synthetase 2 (ACSS2). Finally, mTORC2 activation promotes lipogenesis via AKT-dependent and independent processes, with the latter involving phosphorylation of serum- and glucocorticoid-regulated kinase (SGK1) and Protein kinase C (PKCs), followed by sterol regulatory element binding protein-1c (SREBP1c) activation.

A significant volume of research reveals that metabolic rewiring is caused by changes in oncogenes and tumor suppressors, which are regulated by major metabolic enzyme effectors [57]. The proto-oncogene *c-Myc* has been shown to cause a metabolic shift to glycolysis in tumor cells and to control the majority of glycolytic enzymes [58]. An initial study found that *c-Myc* also activates genes involved in mitochondrial structure and function, as well as promoting mitochondrial biogenesis [26]. Another major oncogene, *HIF-1* is an important regulator in glucose metabolism in cancer cells in a hypoxic environment a hallmark feature of tumorigenesis. Several enzymes of the glycolytic pathway, including glucose transporter 1 (GLUT1), glucose transporter 3 (GLUT3), HK1, HK2, glyceraldehyde-3-phosphate dehydrogenase (GAPDH), phosphoglycerate kinase 1 (PGK1), PKM2, LDH-A, and 3-phosphoinositide-dependent kinase 1 (PDK1), are activated by *HIF-1A*. Moreover, *HIF-1* stimulates glucose absorption and lactate production by inhibiting the TCA cycle and oxidative phosphorylation in the mitochondria [59,60] (Figure 3).

## 4. Role of Tumour Suppressor Genes and miRNAs in Cancer Metabolism

**Figure 4:**
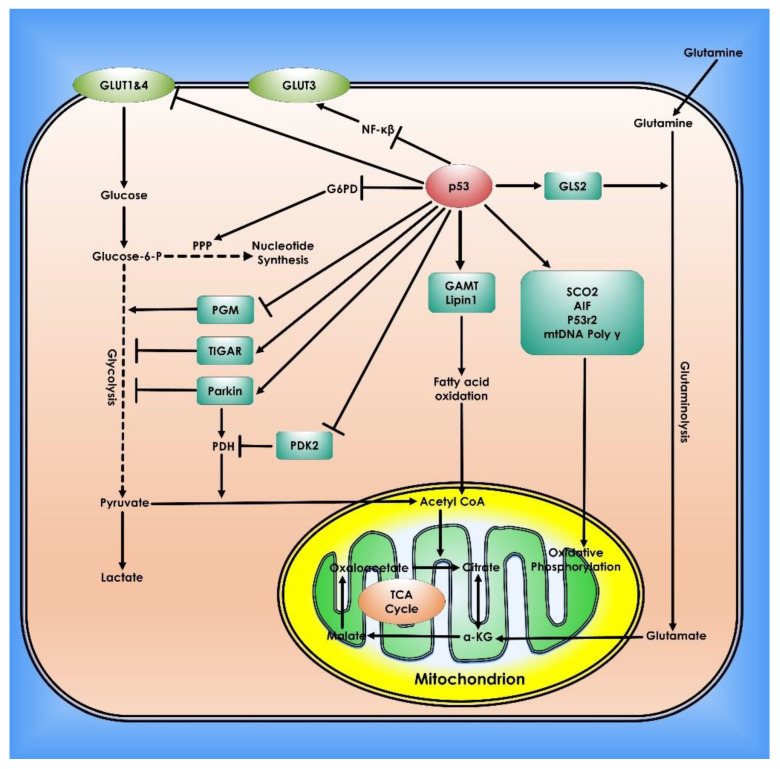
Role of p53 in Cancer Metabolism. In cells, p53 controls mitochondrial oxidative phosphorylation, glycolysis, glutaminolysis, and fatty acid oxidation. To preserve mitochondrial integrity and increase oxidative phosphorylation, p53 transcriptionally stimulates the synthesis Of Cytochrome C Oxidase 2 (SCO2), Apoptosis-inducing Factor (AIF), and p53-controlled ribonucleotide reductase (p53R2), and physically interacts with mtDNA Poly. p53 inhibits glucose absorption through direct suppression of GLUT 1 and 4 transcription and indirect repression of GLUT 3 expression. p53 inhibits glycolysis via adversely regulating phosphoglycerate mutase (PGM) at the protein level and transcriptionally inducing *TIGAR* and Parkin. PDH, which converts pyruvate to acetyl-CoA, is favorably regulated by Parkin. p53 suppresses the production of PDK2, which phosphorylates and inhibits the function of PDH. Glutaminase 2 (GLS2) catalyzes the hydrolysis of glutamine to glutamate and is induced by p53. This can then be transformed into α-KG. GLS2 enhances the TCA cycle and oxidative phosphorylation by raising α-KG levels. p53 physically interacts with G6PD to inhibit its activity, hence inhibiting PPP, a crucial mechanism for nucleotide synthesis and NADPH generation. p53 promotes fatty acid oxidation by inducing the expression of Guanidinoacetate methyltransferase deficiency (GAMT) and Lipin1. Fatty acid oxidation helps the maintenance of the TCA cycle and mitochondrial oxidative phosphorylation by generating acetyl-CoA.

Numerous studies have demonstrated the pivotal role of protein-coding tumor suppressor genes, as well as regulatory noncoding small miRNAs in normal and cancer cell metabolism. The master regulator and tumor-suppressor p53 decrease glucose absorption in cells by downregulating GLUT1 and GLUT4 expression in cancer cells. Hence, it plays a vital role in suppressing glycolysis under normoxic or hypoxic conditions via its transcriptional target genes [59,60]. The DNA Binding Domain (DBD) especially the amino acid residues 98–293, which is located in exons 5, 6, 7, part of exons 4, and 8 of *p53*, contains the majority of the high-frequency oncogenic mutations, as well as an aggregation-prone region (APR), and has been proven in multiple studies to be a useful model system to emulate the behavior of full-length p53, including aggregation [61,62]. Expression of murine double minute 2 (Mdm2) can also be targeted by the activation of a ROS-ERK2-MDM2 axis in cancer cells by destabilizing mutant *p53* gene, the p53 negative regulator, is commonly amplified and/or overexpressed in numerous cancers, resulting in p53 signaling failure [61]. *TP53*-induced glycolysis and apoptosis regulator (TIGAR) a p53-transcriptional target gene reduces fructose-2,6-bisphosphate levels in cells and slows glycolysis. Interestingly, *TIGAR* operates through the PPP to reduce intracellular ROS levels [63]. Recent research shows that sirtuin-family deacetylases (SIRT) play key roles in the regulation of metabolism, therefore, affecting cancer development. It has been shown that SIRT1, SIRT2, SIRT3, SIRT4, or SIRT6 deletion causes tumor development in an animal model of cancers [64]. Sirtuins appear to tune glucose metabolism via modulating *c-Myc* and *HIF-1*, two important factors of cancer metabolic remodeling. Importantly sirtuins affect the activity of glycolytic enzymes through deacetylation [63,64,65,66]. SIRT3, a mitochondrial deacetylase, enhances mitochondrial metabolism by deacetylating and activating the TCA cycle and fatty acid oxidation enzymes [67] (Figure 4).

**Figure 5:**
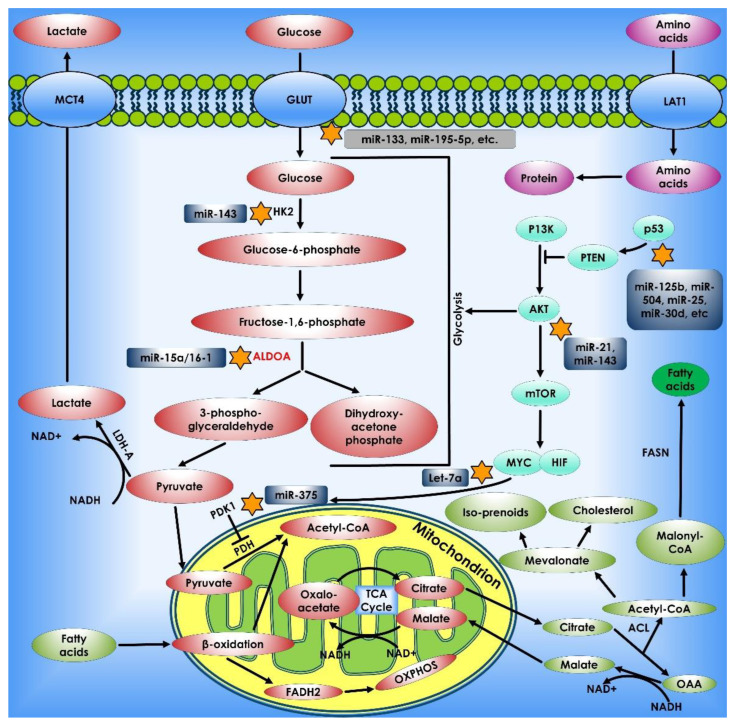
miRNAs and Cancer Metabolism. miRNAs control cell metabolism by targeting critical metabolic enzymes and oncogenic signaling pathways. miRNAs (shown in the dark blue shaded boxes) potentially influence cell metabolism via altering the expression of metabolic transporters (such as GLUT) or enzymes (such as HK2, ALDOA, and PDK1), as well as by acting on the p53, *c-Myc*, and AKT/mTOR signaling pathways.

Evidence is growing that miRNAs have a role in the control of the Warburg effect, through interactions with oncogenes/tumor suppressors including *c-Myc*, *HIF-1*, and p53. Prostate cancer gene expression marker 1 (PCGEM1), a long noncoding RNA (LncRNA), has lately been found to increase glucose absorption for aerobic glycolysis by *c-Myc* activation [68,69] Many cancers, including lymphoma and colorectal cancer, have high levels of the miR-17-92 cluster miRNAs, which are transcribed as a polycistronic unit. They can enhance the oncogenic action of *c-Myc* and so act as an oncogene [70,71,72]. In lung and liver tumors, miR-221 and miR-222 are typically overexpressed. Their overexpression has been shown to increase tumorigenicity by inhibiting the tumor suppressors phosphatase and tensin homolog, and tissue inhibitor of metalloproteinase 3 [73]. miR-504 is a new miRNA that can suppress p53 production by binding to two binding sites in the human p53 3′-UTR. The results show that ectopic expression of miR-504 decreases p53 protein levels and inhibits p53 activities, particularly p53-mediated apoptosis and G1 cell cycle arrest in stressed cells [74]. According to one research, miR-125b is another new miRNA that targets p53. miR-125b is a brain-enriched miRNA that functions as a p53-negative regulator in both zebrafish and humans. Overexpression of miR-125b suppresses endogenous p53 protein levels and decreases apoptosis in cells, whereas knockdown of miR-125b enhances p53 protein levels and causes apoptosis in human cells and the zebrafish brain [75] (Figure 5).

## 5. *c-Myc* and Cancer Metabolism

The revelation that LDH-A, which converts pyruvate to lactate as part of the glycolytic pathway, was one of twenty potential *c-Myc* target genes was the first indication that *c-Myc* played an essential role in glycolysis regulation [26,76,77]. Further research has revealed that *c-Myc* regulates numerous additional glucose metabolism genes as well [78]. *GLUT1, HK2, PFKM*, and enolase 1 (ENO1) are among the genes involved [79,80,81]. *c-Myc* directly contributes to the Warburg effect (aerobic glycolysis) and the capacity of transformed cells to convert glucose to pyruvate even under appropriate oxygen tension by up-regulating these genes. *ENO1* has been demonstrated to give rise to another translation initiation product, *c-Myc* promoter-binding protein-1 (MBP-1), which is a negative regulator of *c-Myc* expression [82]. This creates a negative feedback effect that is regulated by hypoxia [83].

Transgenic animal studies have validated the immediate effects of *c-Myc* expression on glycolytic capability [84]. Mice with *c-Myc* overexpression in the liver have enhanced glycolytic enzyme activity and create more lactic acid. Stably transfected mouse fibroblasts overexpressing LDH-A alone or those transformed by *c-Myc*, on the other hand, overproduce lactate. This shows that LDH-A, a downstream target of *c-Myc*, can cause the Warburg effect. By reducing LDH-A expression, the soft agar clonogenicity of Burkitt’s lymphoma cells is significantly reduced [26].

*c-Myc* is vital in mitochondrial biogenesis in addition to its involvement in regulating cellular metabolism through modulating the expression of genes involved in metabolic pathways. Large-scale gene expression investigations in rat and human systems originally revealed that *c-Myc* overexpression might activate nuclear-encoded mitochondrial genes [85,86,87]. Furthermore, *c-Myc* has been found to bind to the promoters of genes encoding mitochondrial proteins [85,88]. It was demonstrated that mitochondrial biogenesis is reliant on *c-Myc* expression using an inducible *c-Myc*-dependent human B cell model of cell proliferation [26]. Furthermore, mitochondrial biogenesis genes were among the most strongly elevated *c-Myc* target genes.

*c-Myc* appears to improve mitochondrial function in addition to its involvement in mitochondrial synthesis. It has been demonstrated that *c-Myc* stimulates mitochondrial acetyl-CoA production, which contributes to large increases in histone acetylation and fatty acid biosynthesis in rapidly proliferating cells [89,90]. Mitochondria not only serve as a means for efficient production of ATP in the presence of oxygen, but they also play an essential role in generating substrates for macromolecular synthesis in dividing cells. The ability of *c-Myc* to induce mitochondrial biogenesis in proliferating cells while impeding mitochondrial respiration is critical. Pyrimidines, whose production is directly related to the electron transport chain, the carbon backbone for amino acids, and citrate, which is converted to acetyl-CoA for lipid biosynthesis are among these components. These roles supplement *c-Myc*’s stimulation of glucose uptake and metabolism, which provides carbon backbones for essential cellular components such as ribose for nucleotide biosynthesis and NADPH for redox balance via the PPP, triglycerides, and ATP via glycolysis.

Studies have shown that *c-Myc* expression has a considerable effect on cancer-related changes in glucose and glutamine metabolism [26]. The observation of persistent, *c-Myc*-dependent hypoxic glutamine metabolism even in the absence of glucose shows that *c-Myc* expression has a significant impact on this. Overexpression in transformed cells resulted in the simultaneous metabolism of glucose to lactate and the oxidation of glutamine via the TCA cycle [26]. Under hypoxic circumstances with elevated *c-Myc*, a significant portion of the glucose eaten was converted to expelled lactate, whereas glutamine was utilized by the TCA cycle for cell survival. This study also discovered that under glucose-depleted culture conditions, a glutamine-dependent and glucose-independent TCA cycle may occur under both aerobic and hypoxic circumstances. Furthermore, they discovered an increase in glutamine to glutathione conversion under hypoxia; glutathione is a key reducing agent for limiting the buildup of mitochondrial ROS. They also showed that inhibiting glutaminase kills hypoxic cancer cells in vitro and slows tumor xenograft development.

**Figure 6:**
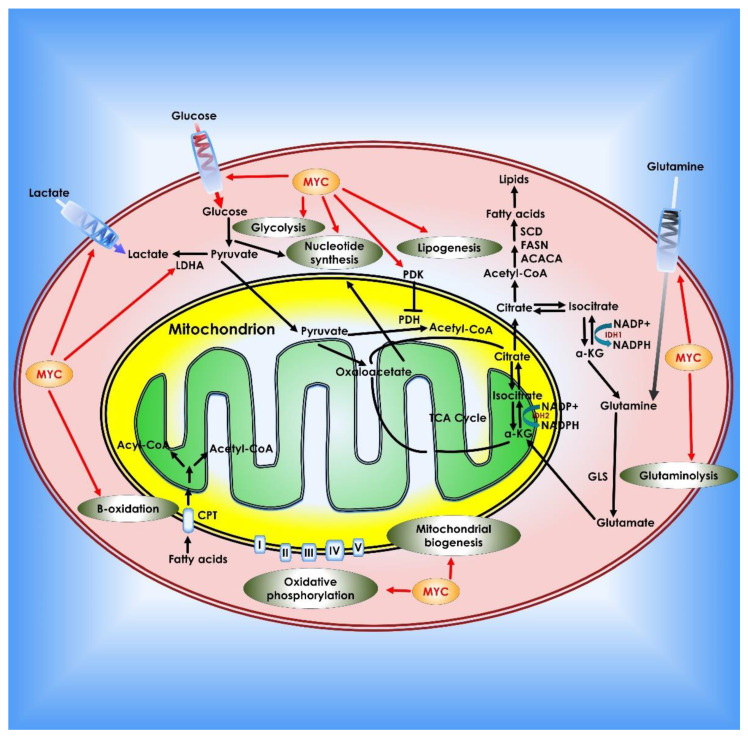
Role of *c-Myc* in cancer metabolism. *Myc* contributes to tumor cell metabolic reprogramming on several levels. *Myc* enhances the Warburg effect by boosting glucose absorption by up-regulation of glucose transporters, increasing glycolysis and lactate generation (via regulation of LDH-A and the lactate transporter MCT1). *Myc* also regulates pyruvate dehydrogenase, which prevents pyruvate from entering the TCA cycle. *Myc* promotes glutamine addiction in cancer cells by upregulating glutamine transporters and suppressing the production of miRNA-23a/b, which inhibits GLS1. *Myc* stimulates fatty acid synthesis by modulating numerous lipogenesis enzymes such as acetyl-CoA carboxylase (ACACA), FASN, and stearoyl-CoA desaturase (SCD). *Myc* also promotes nucleotide synthesis, mitochondrial biogenesis, fatty acid β-oxidation, and oxidative phosphorylation.

Interestingly, *c-Myc* appears to influence glutamine metabolism by suppressing the miRNAs miR-23a and miR-23b [26,91]. As a result, the expression of their target protein, mitochondrial glutaminase, increases. This, in turn, causes an increase in glutamine catabolism, resulting in more glutamate, which is then metabolized via the TCA cycle or acts as a substrate for glutathione formation [92]. This surprising gene regulatory system links *Myc* control of miRNAs, glutamine metabolism, and ROS homeostasis (Figure 6).

## 6. Fatty Acid Metabolism in Cancer Cells

Higher fat production has lately been established as another major distortion of metabolism requisite for carcinogenesis while garnering less emphasis than aerobic glycolysis [93]. FA production is generally enhanced in cancer cells to meet the lipid need for membrane and signaling molecule synthesis, and cancer cells typically accumulate more lipids in the form of lipid droplets than normal cells. Citrate is synthesized in the mitochondria via the TCA cycle and then transported across the inner mitochondrial membrane into the cytosol by the transport protein citrate carrier (CIC), where it is used in de novo FA production. CIC activity is necessary for tumor proliferation in vitro and carcinogenesis in vivo, and its levels are raised in human cancer cell lines [94].

In de novo lipogenesis, ACLY functions as a primary rate-limiting enzyme. It links glucose and FA metabolism by converting citrate to oxaloacetate and acetyl-CoA, which is used in FA synthesis in the cytoplasm, by converting glucose-derived citrate into acetyl-CoA. ACLY upregulates histone acetylation in mammalian cells in response to growth factor stimulation [90]. ACLY expression is highly upregulated in colorectal cancer, breast cancer, glioblastoma, and ovarian cancer suggesting its role in the metabolic activities of different cancers. ACLY upregulation enhances tumor cell development, whereas ACLY silencing decreases tumor cell growth [95,96,97,98]. Acetylation of ACLY stabilizes it by preventing ubiquitylation and degradation, and it stimulates de novo lipid synthesis and tumor cell proliferation, whereas deacetylation of ACLY by a deacetylase, such as SIRT2, destabilizes it [99]. ACLY has been shown to influence the cell cycle in addition to its major role in de novo lipogenesis. ACLY inhibits AMP-activated protein kinase (AMPK) activity by directly interacting with the catalytic domain of AMPK, and activation of AMPK in the absence of ACLY may result in p53 activation, ultimately leading to cellular senescence.

Acetyl-CoA carboxylase (ACC) catalyzes the carboxylation of acetyl-CoA to malonyl-CoA, another rate-limiting step in fatty acid synthesis [100]. The human genome has two types of ACC, ACC1, and ACC2. ACC1 is found in the cytosol and is predominantly expressed in lipogenic tissues, whereas ACC2 is found in the mitochondrial membrane and is found largely in oxidative tissues [101]. ACC1 is in charge of the rate-limiting phase of de novo fatty acid synthesis, converting acetyl-CoA to malonyl-CoA. ACC2 is suspected to play a role in the regulation of fatty acid oxidation rather than fatty acid production. AMPK, a critical metabolic sensor that is directly phosphorylated and activated by the tumor suppressor LKB1, significantly reduces fatty acid synthesis by phosphorylating and inactivating ACC1 [99].

**Figure 7:**
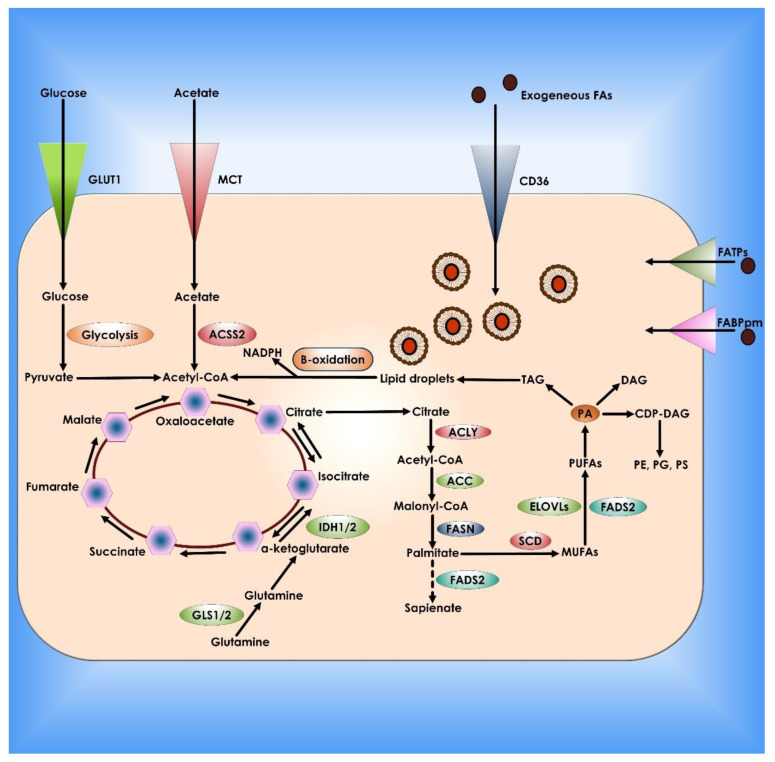
Lipid metabolism in Cancer Cells. De novo lipogenesis and exogenous absorption provide fatty acids (FAs) to cancer cells. Specialized transporters, including CD36, FATPs, and FABPpm, allow external absorption of FAs from the surrounding milieu. FAs and their synthetic products can then be stored as LDs and utilized to produce NADPH and acetyl-CoA via oxidation. Cancer cells use glucose, glutamine, and acetate to synthesize citrate as carbon sources for de novo lipogenesis. Palmitate is formed from citrate by the enzymatic activities of ACLY, ACC, and FASN and can then be desaturated and extended to create a broad set of lipid species. There is an alternate mechanism for palmitate desaturation that produces sapienate rather than palmitoleate via FADS2.

Malonyl-CoA decarboxylase (MCD) catalyzes the conversion of malonyl-CoA to acetyl-CoA and carbon dioxide. Malonyl-CoA levels in the cell alter the balance of FA synthesis and oxidation. SIRT4 binds, deacetylates, and inhibits malonyl-CoA decarboxylase as the mitochondrial sirtuin MCD. As a result, SIRT4 inhibits FA oxidation while boosting lipid anabolism [102]. The final stages in the de novo synthesis of FA are catalyzed by FASN. Palmitate was generated from malonyl-CoA and acetyl-CoA substrates by sequential condensation processes in the presence of NADPH and then processed into saturated fatty acids. FASN is overexpressed in many forms of cancer, and increased FASN expression and activity confer a survival advantage in cancer cells [103]. According to research, FASN is a metabolic marker of cell proliferation rather than a hallmark of malignancy in ovarian cancer and its precursor cells. Although the absence of FASN expression in quiescent normal cells indicates that it is a promising target for future therapeutic development [104]. SREBP1, a transcription factor and a member of the basic helix loop helix leucine zipper (bHLHLZ) family, regulates lipogenic processes by activating a varied range of genes involved in fatty acid and triglyceride syntheses, such as ACLY, ACC1, and FASN [102]. SIRT6, an NAD+-dependent protein deacetylase, inhibits SREBP1 expression and activity, as well as transcription of its target genes, resulting in decreased triglyceride levels in the hepatocytes [103]. SIRT1, however, promotes endometrial tumor formation by upregulating SREBP1 expression, consequently enhancing lipogenesis [104]. It was shown that mTORC1 regulates SREBP1 activity and contributes to Akt-dependent lipogenesis and cell proliferation. However, a new study discovered that mTORC2 also serves as a critical signaling hub for FA metabolism, and this is accomplished by activating downstream AGC kinases such as AKT, serum- and glucocorticoid-regulated kinases (SGKs), and PKCs [105,106] (Figure 7).

## 7. Enzyme of the Branched Chain Amino Acid Metabolism in Cancer Cells

**Figure 8:**
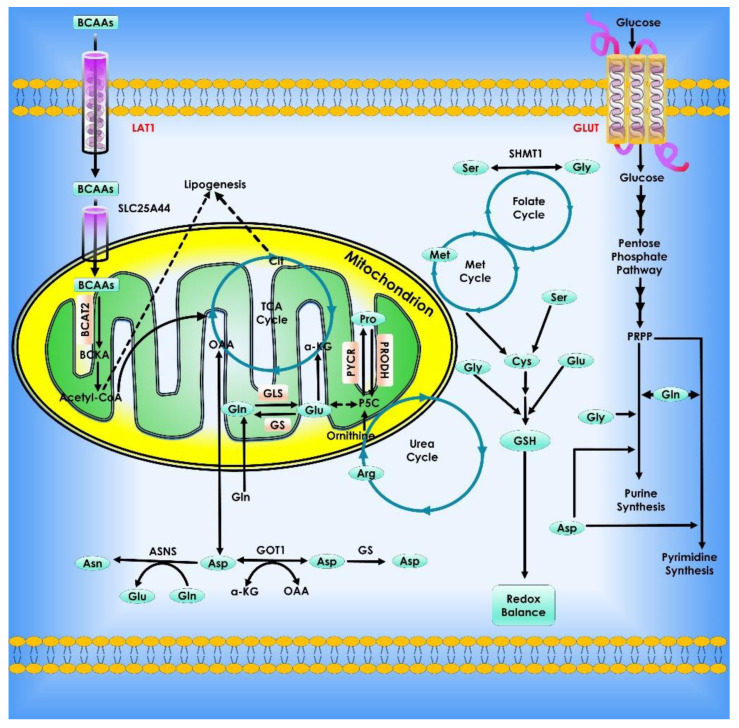
Amino acid metabolism in cancer cells. Cancer cell growth and proliferation rely heavily on metabolic reprogramming. Both essential and non-essential amino acids (EAAs and NEAAs) contribute to altered metabolism by acting as energy sources, biosynthetic agents, and redox balance mediators. Amino acids provide metabolic intermediates like acetyl-CoA, which fuel energy generation via the citric acid cycle. Amino acids also serve as the foundation for nucleotide synthesis and lipogenesis, both of which are essential for a cell’s capacity to grow and develop. To counteract the consequences of oxidative stress, amino acids can control redox equilibrium by producing glutathione. Furthermore, EAA catabolism contributes to the production of NEAAs by chemical processes such as those mediated by transaminases.

Branched-chain amino acids (BCAA) metabolism remodeling is determined by changes in the expression and activity of BCAA transporters and metabolic enzymes involved. Branched-chain amino acid aminotransferase (BCAT) is controlled by oncogenes and tumor suppressors in cancer cells, resulting in carcinogenesis. Several transcriptional regulators, including *c-Myc, HIF-1*, and *SMAD5*, have binding sites in *BCAT1*’s promoter region. *HIF-1* has been shown to upregulate both the mRNA and protein levels of *BCAT1* in human glioblastoma cell lines and primary glioblastoma spheres in hypoxia circumstances [105,106,107,108]. Although both *HIF-1* and *HIF-2* may directly bind to the hypoxia response element in the first intron of the *BCAT1* gene, only *HIF-1* is functional in activating *BCAT1* transcription. Similarly, *HIF-1* and *HIF-2* increase the BCAA transporter large neutral amino acid transporter (LAT1) in human glioblastoma cells under hypoxia, while HIF-2 is responsible for *LAT1* expression in ccRCC cells [109]. Hypoxia and *HIF* did not affect *BCAT2* expression in glioblastoma. The metabolic tracing experiment revealed that hypoxia promotes nitrogen transfer from BCAAs to glutamate, which is inhibited by *HIF-1/2* deletion, implying that *HIF* is a critical regulator of BCAA metabolic remodeling in human glioblastoma cells in response to hypoxia [110]. Musashi2 (MSI2) was shown to bind to MSI binding sites in the 3′ untranslated region of *BCAT1* in human chronic myeloid leukemia cell lines and positively regulate *BCAT1* transcription. MSI2 is inappropriately active in various human malignancies such as glioma and breast cancer. *SMAD5* is translocated into the nucleus and interacts with the *BCAT1* promoter to stimulate *BCAT1* expression in cancer-associated fibroblasts from pancreatic ductal adenocarcinoma (PDAC) tumors when the transforming growth factor is activated [108,111]. *BCAT2* transcription is activated in pancreatic cancer cells by the transcription factor sterol regulatory element-binding protein 1 (SREBP1) [112]. Furthermore, the mutant Kirsten rat sarcoma viral oncogene homolog (*KRAS*) oncogene controls the increase of *BCAT2* expression at the post-translational level. In *KRAS*-mutant PDAC, the spleen tyrosine kinase SYK is downregulated, resulting in lower phosphorylation of tyrosine 228 on BCAT2 protein, limiting Tripartite motif containing-21 mediated ubiquitination and consequent protein degradation of BCAT2 [113]. The expression of genes involved in BCAA metabolism has been demonstrated to be controlled by several nuclear receptors. Through several nuclear receptors, the peroxisome proliferator-activated receptor (PPAR) coactivator 1 (PGC-1) can promote *BCAT2* and branched-chain keto acid dehydrogenase E1 subunit alpha (BCKDHA) expression in transgenic mice but not branched-chain ketoacid dehydrogenase kinase (BCKDK) expression [114]. Bioinformatics investigation revealed that transcription factors including PPAR and Krüppel-like factor (KLF) 4 are enriched in *BCAT2* and/or *BCKDHA* genes in PGC-1 transgenic mice. By binding to *BCAT2*’s promoter, the glucocorticoid receptor-KLF15 axis co-activates its expression in rat muscle cells [115]. In the rat liver, the peroxisome proliferator-activated receptor (PPAR) is thought to activate the BCKDH complex by downregulating BCKDK. The expression of PPAR in muscle, liver, and white adipose tissue was increased in BCAA-fed mice, showing a feedback loop between nuclear receptors and BCAA metabolism [116,117,118]. DNA methylation at *BCAT1*’s promoter inhibits transcription, which is linked to *BCAT1* downregulation in isocitrate dehydrogenase (IDH) mutant anaplastic astrocytoma and glioblastoma. In hepatocellular carcinoma, methylation of the *BCAT1* promoter is decreased, resulting in higher *BCAT1* expression [119]. G9a and SUV39H1 are histone modifiers that catalyze di- and tri-methylation of histone H3 (H3K9) at the promoter of the *BCAT1* gene, resulting in *BCAT1* downregulation in lung cancer cells [120]. Similarly, in leukemia, EZH2, the catalytic member of the polycomb repressive complex 2 that promotes H3K27 methylation, inhibits *BCAT1* expression [121]. The oncometabolite R-2-hydroxyglutarate (2-HG), which is generated by mutant IDH1/2 in glioma and competes with α-KG for binding, inhibits the transaminase activity of *BCAT1* and *BCAT2*. Oncogenic factors and tumor suppressors work together to fine-tune the expression levels of *BCAT* and BCKDH, causing BCAA metabolic reprogramming [122] (Figure 8).

## 8. Glutamine Metabolism in Cancer Cells

Upregulation of the glutamine metabolism is a signature metabolic alteration in carcinogenesis. Interestingly, glutamine is the second most important nutrient after glucose in multiple cancer types. Glutamate contributes nitrogen and carbon to several activities in cancer cells, including energy generation, macromolecular synthesis, and signal transmission. Transporters (such as SLC1A5 or ASCT2) carry glutamine into the cytoplasm, and glutaminase (GLS) facilitates glutamine catabolism by converting glutamine to glutamate [123,124]. The oncogenic transcription factor *c-Myc* enhanced glutaminase production and glutamine metabolism in cancer cells [26,125]. Master tumor suppressor p53 directly binds to GLS2 and transcriptionally modulates its expression affecting glutamine metabolism and GSH antioxidant activity and consequently lower levels of intracellular ROS [126]. Glutamate can be carried into mitochondria and converted into α-KG by oxidative deamination by GLUD1. Transamination of glutamate to α-KG can occur in the cytoplasm or mitochondria, resulting in the production of non-essential amino acids (e.g., serine). Following that, the mitochondrion’s α-ketoglutarate is used as a TCA cycle intermediary for energy recycling. To manufacture AcCoA for lipid synthesis, human cells use reductive metabolism of α-KG. Under normal culture circumstances, this IDH1-dependent process is active in most cell lines, whereas cells grown under hypoxia depend nearly entirely on the reductive carboxylation of glutamine-derived α-KG for de novo lipogenesis. Moreover, when glucose is depleted, glutamine-derived fumarate, malate, and citrate were dramatically elevated, suggesting that glutamine drives the glucose-independent TCA cycle. Many tumor cells have increased glutamine usage for mitochondrial-dependent bio-energy generation and cellular biosynthesis. Silencing of a tumor suppressor, which is also a kinase B1, a serine/threonine kinase leads to increased glucose and glutamine consumption in tumor cells. The kinase B1 appears to link bioenergetics processes to cell growth control via the regulation of mTOR activity [127]. Many malignant cells utilize acetyl-CoA, which is mostly synthesized from the glucose metabolism product pyruvate, whereas glucose deprivation triggers a cascade in which acetyl-CoA is produced from glutamine via GDH. Inhibiting mitochondrial pyruvate transport, on the other hand, activates GDH which modulates glutamine metabolism to create oxaloacetate and acetyl-CoA, resulting in lipid synthesis that is glutamine-dependent [128].

In addition to the extracellular absorption, Serine is also produced from the glucose metabolism pathway. Interestingly, Serine and glycine biosynthesis which appears to be connected produces precursors essential for the synthesis of proteins, nucleic acids, and lipids. These macromolecules in turn are essential ingredients for cancer development. In this context, it is important to note that *SHMT*s genes, which are direct *c-Myc* transcriptional targets, may catalyze both de novo serine generation from 3-phosphoglycerate and imported serine conversion to glycine [129]. Threonine dehydrogenase and glycine C-acetyltransferase can also convert threonine to glycine. Glycine then offers methyl groups for one-carbon metabolism, which is essential for nucleic acid, protein, and lipid production, as also DNA methylation [130].

**Figure 9:**
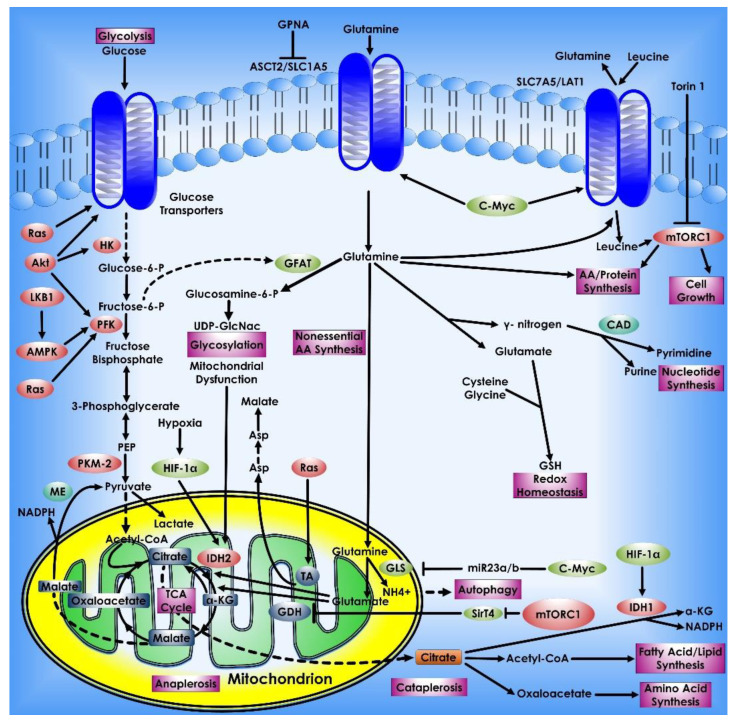
Glutamine metabolism in Cancer Cells. Glutamine is a key metabolic fuel that assists rapidly growing cells in meeting the increased need for ATP, biosynthetic precursors, and reducing agents. Glutamine enters the cell via the amino acid transporter ASCT2/SLC1A5 and is transformed into glutamate in the mitochondria via a glutaminase-catalyzed deamination process (GLS). Glutamate is metabolized to the TCA cycle intermediate α-KG by GDH or alanine or aspartate transaminases (TAs), which also create the respective amino acid. α-KG is an important molecule that aids in both ATP generation and the replenishment of TCA cycle intermediates, a process known as anaplerosis. α-KG can be converted to citrate in a reductive carboxylation process mediated by IDH2 during periods of hypoxia or mitochondrial malfunction. The freshly generated citrate leaves the mitochondria and is utilized to manufacture fatty acids and amino acids as well as the reducing agent, NADPH (cataplerosis). In the cytosol, glutamine lends its (amide) nitrogen for the synthesis of nucleotides and hexosamines, resulting in the production of glutamate. Through the synthesis of glutathione, cytosolic glutamate is essential for maintaining redox equilibrium and protecting cells from oxidative damage. Many cancer cells have oncogene-dependent glutamine addictions, and glutamine can increase proliferative signaling. For example, glutamine influx via *SLC1A5* is linked to efflux via the SLC7A5/LAT1 transporter, enabling leucine into the cell and activating mTORC1-mediated cell growth. Furthermore, the signaling molecules Akt, Ras, and AMPK stimulate glycolytic enzymes and promote lactate synthesis (Warburg effect), requiring cancer cells to use glutamine metabolism to fulfill higher energy needs. The proto-oncogene *c-Myc* promotes glutaminolysis by activating the *GLS* and *SLC1A5* genes through transcriptional activation. Proteins that have been glycosylated by glutamine, including growth factor receptors, can be targeted to the cell surface and activated.

Proline is a one-of-a-kind proteinogenic secondary amino acid that is stored in collagen, the body’s most ubiquitous protein [131]. Proline and glutamate are interconvertible, using D1-pyrroline-5-carboxylate (P5C) and glutamic-c-semialdehyde (GSA) as intermediates. Proline Dehydrogenase/Proline Oxidase is a mitochondrial tumor suppressor that catalyzes the conversion of proline to P5C. It is activated by p53 and PPARc but repressed by miR-23b* and *c-Myc* [123]. In the Urea cycle, GSA generated from glutamate or proline can be converted to ornithine, which is a prerequisite for arginine production [131]. Arginine is an important amino acid, and arginine deficiency caused several types of solid tumor cells to die quickly in culture media [124]. Arginine is involved in several essential cellular metabolic processes, including the urea cycle, nitric oxide biosynthesis, nucleotides, proline, and glutamate biosynthesis [125]. The rate-limiting enzyme for the de novo production of arginine is argininosuccinate synthetase (ASS), which catalyzes the synthesis of argininosuccinate from L-citrulline and aspartic acid. Following that, argininosuccinate lyase (ASL) transforms argininosuccinate into L-Arginine and fumaric acid, the latter of which connects arginine metabolism to glucose-generated energy metabolism via the TCA cycle. Some human malignancies, particularly malignant melanoma and hepatocellular carcinoma, lack ASS and so are vulnerable to arginine deprivation treatment employing arginine-degrading enzymes because they are unable to generate arginine [125]. Recombinant arginine-degrading enzymes were employed to treat this type of tumor since these cancer cells would die if they were exposed to them (arginine deiminase or arginase) (Figure 9).

## 9. Targeting Cancer Metabolism for Therapeutic Purposes

Cancer metabolism is an extremely promising and quickly expanding therapeutic avenue in the current landscape of anti-cancer drug research due to its crucial part in tumorigenesis, which is caused by metabolic reprogramming. The selective and effective inhibition of tumor-relevant metabolic enzymes is now possible with a wide variety of new drugs (Table 1). Many factors must be taken into account while deciding on the best metabolic target for cancer therapy. Some metabolic enzymes may be systemically harmful because of their physiological roles in normal tissues [126]. These pathways can be targeted therapeutically if the systemic inhibition of the route is tolerated. As cancer cells alter their metabolism, so do normal proliferating cells, such as immune cells and stem cells [127,128]. The adaptive immune system should be unaffected by metabolic inhibitors. It’s still possible to find outstanding instances of cancer therapies that benefit from the reprogramming of certain circuits. Anti-folates (methotrexate, pemetrexed, and others) have been shown to target tumor cells’ increased nucleotide and DNA synthesis, which is a hallmark of cancer [129]. Cancer treatment regimens are generally effective although these medications might cause toxicity in normal proliferative tissues such as the intestinal epithelium and bone marrow. Because of this, it is important to study the impact on normal cells of metabolic enzyme inhibition. There is a growing body of evidence indicating cancer cells can change their metabolic profile during carcinogenesis and metastasis. As a result, cancer cells may be able to build resistance to a certain metabolic pathway’s blockage by producing alternative protein isoforms or up-regulating compensatory pathways. For this reason, it is important to target numerous metabolic pathways concurrently or to target a specific metabolic system in conjunction with medicines targeting oncogenic or signaling pathways. Some intriguing metabolic targets are highlighted in this section [128].

One of the initial metabolic targets of *Myc* was LDH-A, an enzyme that converts pyruvate to lactate, which is a byproduct of glycolysis [26]. Experiments in xenograft models have revealed that LDH-A suppression can reduce *Myc*-driven malignancies [35,132]. Studies in mice with genetically engineered NSCLC show that suppression of LDH-A causes tumor shrinkage without causing systemic damage [133]. Myeloid leukemia development is also slowed by the genetic ablation of LDH-A [134]. That’s why cancer cells with *Myc* mutations may be a good target for LDH-A overexpression. HK2 is another glycolytic protein that might be used as a therapeutic target. When HK2 is inhibited in preclinical models of genetically altered NSCLC and breast cancer, tumor growth is delayed [35]. Moreover, systemic HK2 deletion in mice has no negative physiological effects on the organisms involved. LDH-A and HK2 are yet to be studied concerning the adaptive immune system. Immune checkpoint inhibitors may work along with LDH-A inhibition to unleash host inflammatory T lymphocytes that will preferentially assault tumor cells, as lactate has been reported to limit T-cell cytotoxicity [135]. Reprogramming macrophages by lactate can potentially be used to induce cancer [136]. Thus, targeting LDH-A or HK2 in cancers that are highly glycolytic and overexpress these proteins may be beneficial.

PHGDH, an enzyme in the de novo serine synthesis pathway, is another glucose-dependent target. Some human melanoma and breast tumors require PHGDH for development in vitro, which is why high levels of PHGDH have been reported [35,137]. The tumorigenicity of p53-null tumors in mice is reduced by the deprivation of the amino acid serine [138]. In the mitochondria, SHMT2 transforms serine into glycine to produce folate intermediates from de novo or exogenous synthesis [139,140]. *SHMT2* expression is high in several forms of cancer and is associated with a poor prognosis. Under hypoxia, the transcription factors *Myc* and *HIF-1* also support survival by inducing *SHMT2*, another transcription factor [141,142]. Targeting PHGDH, SHMT2, or other enzymes in the one-carbon metabolic pathway has not yet been proven to slow or halt tumor development in genetically modified, patient-derived xenografts (PDX), or syngeneic animal models of cancer. Even while one-carbon metabolism is critical for the anabolic demands of tumor cells, this route is likely required for tumor growth in vivo [143]. As a result of the discovery that metformin, an antidiabetic medicine, is also an anticancer agent, mitochondrial metabolism has emerged as a crucial target for cancer therapy [144]. Diabetic people who use metformin to regulate their blood glucose levels are less likely to acquire cancer and have an increased survival rate if cancer is already present, according to several epidemiological studies [145]. Metformin has also been shown to have anti-cancer properties in laboratory experiments [141,146,147]. Reversible inhibition of mitochondrial complex I by metformin has been discovered by biochemists [148,149,150]. Recent research shows that metformin inhibits mitochondrial ETC complex I, which is linked to cancer [151]. Metformin specifically inhibits the generation of mitochondrial ATP, resulting in the death of cancer cells when glycolytic ATP levels drop due to a lack of glucose. Cancer cells’ ability to synthesize lipids, amino acids, and nucleotides is similarly inhibited by metformin, which is found in the mitochondria [152]. Organs such as the liver and kidneys include organic cation transporters (OCTs) that allow metformin to be absorbed, making it safe for long-term use [153]. Metformin can be absorbed by tumor cells that express OCTs [154]. There is no metformin to block mitochondrial complex I in malignancies that do not have OCTs. It is not apparent if the current antidiabetic dose of metformin used in clinical studies is sufficient to inhibit mitochondrial complex I in malignancies. Metformin at greater dosages than those now used for diabetes may thus be more effective and safe. Phenylbiguanide phenformin, like metformin, has anticancer effects due to its ability to suppress mitochondrial complex I activity [155]. Aside from the fact that it is more easily carried into tumor cells than metformin, Phenformin is no longer prescribed to humans for this same reason. Despite this, phenformin should be considered a viable cancer treatment because lactic acidosis can be managed. Serine deprivation or tumors that have lost p53 or LKB1 can increase the susceptibility of mice to biguanide [141,156,157]. Because of this, biguanides and other mitochondrial complexes I inhibitors may be useful anticancer medicines.

Autophagy and glutaminase inhibitors might also be used to decrease mitochondrial metabolism in some cancers. Short-term suppression of autophagy has been demonstrated to reduce tumor development without causing systemic damage in NSCLC mice models. Autophagy produces amino acids, such as glutamine, that feed the TCA cycle in NSCLC and pancreatic malignancies [141,158]. However, in models where tumors are dependent on glutamine for TCA cycle metabolism in the lack of autophagy, the antitumor efficacy of glutaminase inhibitors has been shown [26,159,160]. Acetate metabolism can be targeted as an alternate strategy. Mitochondria provide the cell with acetyl-CoA, but cancer cells can also utilize acetate during metabolic stress (hypoxia or food restriction) to sustain cell growth and survival [161]. Accumulation of acetyl-CoA is not required for proper development, so ACCS2 is an attractive target for acetate metabolism. ACCS2 knockout mice do not show any obvious signs of disease, yet models of hepatocellular carcinoma with ACCS2 genetic deletion experience reduced tumor burden [162]. Inhibitors of the oxidation of acetate may be effective against human glioblastomas [163]. Autophagy and other mechanisms that supply important metabolic intermediates may thus be effective in specific settings.

Due to the ineffectiveness of single-agent mitochondrial inhibitors, combined treatment is likely the best option. The combination of metformin and current therapeutic PI3K inhibitors, which restrict glucose uptake and glycolysis, is an example of a strategy that might affect both sources of ATP within cells [164]. Although most cancer cells are killed by treatments targeting oncogenes such as *KRAS, BRAF,* and *NOTCH1*, resistant cells that are more sensitive to inhibitors that disrupt mitochondrial metabolism are formed [165,166,167]. Increased mitochondrial inhibitor sensitivity in cancer-initiating cells provides more evidence that blocking mitochondrial metabolism may limit tumor recurrence [168,169]. Additionally, cancer cells enhance their antioxidant ability to counteract the increased ROS generation that occurs during growth and metastasis [170]. To combat cancer, one additional treatment option is to inhibit cancer cells’ ability to scavenge antioxidants, hence increasing ROS levels and leading to cancer cell death [141]. Multiple antioxidant defense mechanisms rely on the reducing equivalent NADPH. To generate NADPH in the cytosol, the oxidative PPP, malic enzyme 1, IDH1, and one-carbon metabolism all contribute. IDH2 and one-carbon metabolism govern mitochondrial NADPH production in part. Many of these NADPH-producing mechanisms are essential for cell survival and function. Two NADPH-generating systems, however, might be therapeutic targets in the future. G6PDH, an enzyme in the oxidative PPP, transforms NADP+ to NADPH in 400 million persons globally. This system, however, serves as a key source of NADPH for some malignancies, therefore it may be therapeutic to inhibit this process and produce oxidative stress to reduce tumor development. NADPH-producing mitochondrial one-carbon metabolism protein methylenetetrahydrofolate dehydrogenase (NADP+ dependent) 2 (MTHFD2) was shown to be substantially expressed in 19 distinct cancer types, but not in normal adult proliferating cells [143]. In vitro, cancer cells that lack MTHFD2 are more susceptible to oxidant-induced cell death when MTHFD2 is depleted. NADPH depletion and ROS production can be increased by administering large dosages of vitamin C, (ascorbate), sodium-dependent vitamin C transporters absorb vitamin C into cells, while the oxidized form of vitamin C, dehydroascorbate (DHA), is imported by glucose transporters such as GLUT1 into cells [171]. GSH in the cell reduces DHA back to vitamin C, which in turn creates GSSG. GSH is regenerated from GSSG via NADPH-dependent GR thereafter. When vitamin C enters the blood, it is oxidized to DHA, which is then absorbed by cells. By depleting the NADPH and GSH pools and boosting ROS levels, large dosages of vitamin C reduce carcinogenesis in colorectal cancers with oncogenic *KRAS* mutations, which exhibit high amounts of GLUT1 [172]. When used in combination with standard paclitaxel-carboplatin treatment, substantial dosages of vitamin C given intravenously have been shown to be safe in people [173]. Buthionine sulfoximine, an irreversible inhibitor of -glutamylcysteine synthase, which can be safely delivered to people and is beneficial in preclinical tumor models, is another option for reducing GSH levels [174]. It’s also made up of cysteine, glutamate, and glycine; these three amino acids form the tripeptide glutathione. Thus, reducing glutamate levels with glutaminase inhibitors or blocking extracellular cysteine absorption might similarly boost ROS levels in cancer cells to promote cell death.

As stem cells are sensitive to ROS levels, it’s crucial to stratify patients based on their expression levels of a certain antioxidant protein or pathway. Because cancer cells produce a significant amount of ROS, it is important to identify which antioxidant pathways are likely to be activated. Targeting the NRF2 pathway, which is used by many cancer types to maintain redox equilibrium, might be a promising treatment approach [175]. Additionally, NSCLC cells are overexpressed in SOD1 (superoxide dismutase 1), and inhibition of SOD1 kills human NSCLC cells and reduces tumor burden in mice models of NSCLC [175]. Short-term suppression of NRF2 and SOD1 may be an efficient method of killing cancer cells, as these knockout mice grow properly (Table 1).

Nanotechnology has been progressively employed in medicine over the last few decades, including applications for safer and more effective tumor diagnostics, therapy, and targeting. Many advantages of nanoparticle (NP)-based drug delivery systems in cancer treatment have been demonstrated, including excellent pharmacokinetics, specific targeting metabolic components of tumor cells, reduced side effects, and drug resistance [176,177]. NPs utilized in medication delivery systems are often created or selected depending on their size and pathophysiology of the malignancies. Mechanically, nano-carriers in cancer therapy target tumor cells following absorption via the carrier effect of NPs and the positioning effect of the targeted chemical. The medications are then delivered to tumor cells to destroy them [178].

A new glucose oxidase (GOD)-loaded therapeutic vesicular NRs (theraNR) targeted cancer cells by raising tumor oxidative stress and decreasing cancer cell antioxidative capabilities, ablating tumors while generating low systemic damage [179].

Anti-angiogenesis drugs prevent neovascularization, reducing the availability of nutrients and oxygen to the tumor and eventually starving the cancer cells. A therapeutic regime involving nanocarriers loaded with plasmid DNA expressing a human soluble version of vascular endothelial growth factor receptor-1 was carried out in mice bearing pancreatic adenocarcinoma xenografts with promising results [180,181].

Nanomedicine-mediated cancer starvation therapy, as an appealing technique for cancer treatment, might selectively deny nutrition and oxygen supply by antiangiogenesis treatment, tumor vascular disruption or blockage, direct depletion of intratumoral glucose and oxygen, and other mechanisms. Furthermore, two or more therapeutic agents could be easily integrated into a single formulation by incorporating chemotherapeutic drugs, therapeutic genes, enzymes, metal NPs, hypoxia-activated prodrugs, inorganic NPs, Fenton-reaction catalysts, photosensitizers, or photothermal agents, leading to improved treatment outcomes [182].

Recent research suggests that mitochondria might be a potential therapeutic target in cancer. So far, the FDA has authorized only a handful of mitochondria-targeted medications. The ability of medications to reach the target location, with the regulated release of the proper quantities of drug at the correct time, is essential for targeted drug delivery. Mitochondria-targeted nanoparticle platforms (mitoNANO) are a unique family of agents capable of delivering medications to the mitochondria, where they are most required [183].

Oncoproteins can also be targeted for high selectivity, the efficiency of delivery, and potential in vivo performance. A pH-responsive polymeric micelle system was developed in a study to serve as a nanocarrier for the intracellular delivery of a therapeutic protein targeting *c-Myc* [184].

## 10. Conclusions and Challenges

The ability of malignant cells to survive and expand by using conventional metabolic pathways to produce energy, generate biosynthetic precursors, and maintain redox equilibrium is reliant on metabolic reprogramming.

We have witnessed tremendous progress toward understanding the underlying molecular mechanisms and consequences of metabolic reprogramming in cancer. A great deal of data delineates mechanisms of reprogrammed metabolic pathways which are essential for cancer cell growth and proliferation. The current review explains the commonly affected metabolic pathways in cancer and focuses on the crosstalk between metabolic pathways, enzymes, metabolites, and cancer cells and how these can be exploited for potential therapeutic regimes. Chemotherapy and hormone therapy are two commonly used treatments for cancer, but their side effects demonstrate the need for more advanced and specialized techniques. Cancer metabolism is being targeted using a variety of conversion routes, enzymes, and metabolites. Cancer cells have altered metabolic pathways that cause them to proliferate more than normal, which is unusual in differentiated normal cells. *c-Myc*, *HIF-1*, *Ras,* and *PI3K/Akt* are all prominent oncogenes that cause cancer metabolic changes. Major tumor suppressors, on the other hand, such as p53 and LKB1/AMPK, counteract these alterations and regulate cellular metabolism. As a result, cancerous cells can be killed by aiming at these statues. The tumor cells are encouraged to rely on aerobic glycolysis via altering glycolysis, which results in lactate production instead of pyruvate delivery to the TCA cycle. Even though certain tumors have shown an increase in the OXPHOS pathway in comparison to their normal counterparts, this is a big discovery for cancer cells. Uncontrolled cell growth can be combated by selective targeting of the several enzymes in these pathways. Novel reprogrammed metabolic pathways facilitating cancer cells to tolerate intrinsic and extrinsic stress such as nutrient depletion might also be aided by novel tumorigenic mutations resulting in the development of drug resistance.

As a result of targeting cancer-reprogrammed metabolism, a new class of anticancer medications may be developed that may treat a wide range of cancers. Several metabolite analogs are now being explored as possible therapeutic options for tumor metabolism targeting and some have reached a stage of clinical trials as well with promising results. There may be a way to increase the efficacy and reduce the toxicities of potentially strong chemotherapy medications by studying the role of mitochondria in cancer cell metabolism more thoroughly. There is a hope that novel cancer metabolic profiles will lead to the development of a new class of cancer therapies. Because of this, the use of metabolic inhibitors may be a clinically advantageous treatment option.

## Figures and Tables

**Table 1:** Compounds targeting cancer metabolomics: The table lists the most common and recent small molecules, compounds, anti-metabolites, and cytotoxins.

**Compound**	**Target**	**Effect**	**Tumour Types Targeted**
2-deoxyglucose	Hexokinase	Inhibits glycolysis	Advanced solid tumors (e.g., lung, breast, prostate, and gastric)
Lonidamine	Hexokinase	Inhibits glycolysis	Benign prostatic hyperplasia
3-bromopyruvate	Hexokinase	Inhibits glycolysis	N/A
TLN-232	Pyruvate kinase	Inhibits glycolysis	Metastatic melanoma and renal cell carcinoma
Dichloroacetate	PDK1	Reactivates PDH	Metastatic solid tumors, glioma, and GBM
Phenylacetate	Glutamine	Reduces plasma glutamine levels	Brain tumors (e.g., glioma, astrocytoma and medulloblastoma)
Asparaginase and Pegasparaginase	Asparagine	Reduces plasmaasparagine levels	ALL, TCL, and BCL
Arginine deiminase	Arginine	Reduces plasma asparagine levels	Metastatic melanoma and hepatocellular carcinoma
Acetazolamide, Indisulam and other sulfonamides	Carbonic anhydrases	pH regulation	Solid tumors (e.g., pancreatic, lung, melanoma and metastatic breast)
Cariporide	NHE1	pH regulation	N/A
SB-204990	ATP-citrate lyase	Inhibits fatty acid synthesis	N/A
Orlistat, GSK837149A, and C75	FASN	Inhibits fatty acid synthesis	N/A
Temsirolimus and Everolimus	mTORC1	Inhibits mTORC1	Solid tumors (both metastatic and non-metastatic)
Other rapalogues	mTORC1	Inhibits mTORC1	Solid tumors (e.g., pancreatic, endometrial and glioblastoma) and lymphoma
Torin1 and PP242	mTORC1 and mTORC2	Inhibits mTORC1 andmTORC2	N/A
PX-478	*HIF1α*	Inhibits *HIF* signaling	Advanced solid tumors and lymphoma
Acriflavine	*HIF1α*	Inhibits *HIF* signaling	N/A
Tirapazamine and other bioreductivecompounds	Hypoxia	Resensitizes cells to other treatments	Solid tumors (e.g., cervical, SCLC and NSCLC)
Bevacizumab and related compounds	Hypoxia, VEGF and VEGFR	Blocks angiogenesis	Solid tumors (e.g., malignant glioma, NSCLC, ovarian, and colorectal)
MK-0646, BIIB022, AVE1642, and others	IGF1R	Blocks IGF signaling	Solid tumors (e.g., NSCLC, pancreatic, hepatocellular carcinoma and metastatic breast)
BEZ235, XL765, SF1126, and BGT226	PI3K and mTOR	Inhibits signaling from PI3K andmTORC1 andmTORC2	Advanced solid tumors (e.g., malignant glioma and NSCLC)
GDC-0941 and PX866	PI3K	Inhibits PI3Ksignaling	Advanced solid tumors (metastatic breast and non-Hodgkin’s lymphoma)
Perifosine andGSK690693	AKT	Inhibits AKT	Solid tumors (e.g., renal cancer and NSCLC) and lymphoma
Metformin	AMPK andComplex I(mitochondrial)	Activates AMPK	Solid tumors and lymphoma
Antimetabolites (e.g., 5-FU, cytarabine andmethotrexate	Nucleotidebiosyntheticpathway	Inhibits cell proliferation	Many tumor types
